# Kinesiophobia among Breast Cancer Survivors One Year after Hospital Treatment

**DOI:** 10.3390/ijerph192114565

**Published:** 2022-11-06

**Authors:** Ewa Malchrowicz-Mośko

**Affiliations:** Department of Kinesiology, Faculty of Sport Sciences, Poznan University of Physical Education, 61-871 Poznan, Poland; malchrowicz@awf.poznan.pl

**Keywords:** oncology, breast cancer, sport, oncology patient, lifestyle, cancer treatment, kinesiogerontoprophylaxis

## Abstract

Breast cancer (BC) is one of the most dangerous health problems affecting women. Lifestyle-associated determinants like physical activity (PA) play an important role in BC treatment outcomes. Studies suggest that oncology patients are insufficiently physically active. One of the potential barriers is kinesiophobia—fear of movement due to expected pain and fatigue. The aim of this cross-sectional study is to investigate the level of kinesiophobia among women one year after BC hospital treatment depending on socio-demographic variables, stage and type of BC, lifestyle, and comorbidities. Polish women after BC (*n* = 138, age 46.5 ± 9.2, BMI 24.6 ± 4.0) participated in the study and the Tampa Scale of Kinesiophobia (TSK) questionnaire was used in the diagnostic survey. The study results show that women suffer from kinesiophobia after BC. Moreover, every third woman (32.6%) does not practice sport regularly one year after BC treatment. The lifestyle before BC diagnosis impacts the level of kinesiophobia after treatment—women who were not physically active before BC diagnosis declared higher levels than previously active women. The study result shows that a high level of kinesiophobia correlates with a low level of PA among women after BC. Women with obesity and diabetes also declared higher levels of kinesiophobia than women without comorbidities. The type and stage of BC have no influence on the level of kinesiophobia; however, in terms of socio-demographic variables, a direct association between kinesiophobia and age has been found—the greater the age, the higher the level of kinesiophobia. Further research on fear of movement in oncology is required in order to effectively eliminate hypokinetic attitudes in every type of female and male cancer.

## 1. Introduction

The number of breast cancer (BC) survivors increases every year, mainly thanks to the development of new treatments and screening techniques. Physical activity (PA) impacts all the stages of carcinogenesis [1]. There is strong evidence that PA before, during, and after BC diagnosis improves outcomes and that PA protects against recurrence and progression in BC survivors [2], with moderate-intensity recreational activity also decreasing the risk of a new malignant tumor [3,4]. Studies have confirmed the association between regular exercise and BC course and prognosis [5], and the beneficial effects can be achieved even with low exposure. In 2020, the World Health Organization (WHO) issued official indications that adult cancer patients should undertake the same weekly PA as healthy people [6]. Unfortunately, many oncology patients who exercised before their diagnosis did not return to the same habits [7]. The potential of sport as an effective strategy in the management of cancer has gained little attention [8], with survivors being significantly less physically active within their first year after BC diagnosis than they were one year before diagnosis [9,10]. There is evidence for an inverse association between PA and BC, especially for postmenopausal women [11]. PA exerts its greatest benefits on women with BC during or after menopause and among patients with body compositions (body fat, waist circumference, body mass index—BMI) in the upper-normal-to-overweight range [12].

PA after a BC diagnosis may reduce the risk of death from this disease. Studies provide support for reduced overall mortality as well as mortality from BC among patients who engage in PA after BC diagnosis [13,14]. Secondary prevention is of great importance in the cancer survivor population given the substantial medical risks they face [15]. In addition to genetic and environmental factors, modifiable factors such as lifestyle, including an insufficient level of PA and poor or improper diet, also affect the recurrence of BC; it is estimated that 9 in 10 cases of BC are due to non-genetic factors, and approximately 25% of total BC cases should be preventable only by lifestyle interventions [16]. Modern women are often characterized by low PA, high stress levels, poor nutrition, smoking, alcohol consumption, late motherhood, or childlessness—these factors are considered significant for the progression of BC [5]. Obesity plays an important role in particular in the etiology of hormone-dependent breast cancers [17], and obesity and weight gain are negative prognostic factors for BC survival. PA prevents weight gain and may decrease obesity [10].

It is crucial to maintain, promote, and improve the mental and physical condition of women after BC and thus their quality of life. The benefits of PA are observed in secondary prevention among women who undergo cancer treatment and in those who completed such therapy. A significant improvement in the circulatory and respiratory systems is reported [5]; however, unfortunately, studies suggest that many cancer survivors are insufficiently physically active—older participants, women, and overweight or obese participants had significantly lower moderate-to-vigorous PA than their younger male normal-weight counterparts [2]. Regular exercise may be an effective intervention in reducing the state of anxiety in BC survivors, especially those with a high state of anxiety [18]. Exercise in BC survivors has been linked to reductions in cancer-related fatigue, nausea symptoms, and improvements in immune system function [7,19].

Women should try to maintain movement after BC surgery and return to normal use of the associated arm. Unfortunately, some women think that they have to protect their arm from activities because of the risk of lymphedema [20]. Lymphedema is a chronic and progressive long-term adverse effect of BC treatment commonly defined by swelling of the affected arm [21]. BC survivors with decreased PA are at an increased risk of upper extremity lymphedema, and PA and exercise are important to improve lymphatic drainage via the muscles’ contracting [22]. 

PA is, therefore, important for people who have cancer and chronic comorbidities. Unfortunately, people with osteoporosis (which is a common complication of antihormonal BC treatment) have a higher level of kinesiophobia than healthy people [23], while BC and cardiovascular disease are conditions that are directly correlated [24]. In the study conducted by Hair et al. [25], only 35% of respondents met PA guidelines after diagnosis with BC, and a decrease in activity after diagnosis was reported by 59% of patients.

The main barriers to participation in PA are lack of information about the permissible types of activity, bad mood, depression due to diagnosis, apathy, or kinesiophobia (fear of movement) [16]. The literature shows that a high level of kinesiophobia correlates with a low level of PA [26]. Low PA can lead to functional decline, low quality of life, and high mortality, particularly in older cancer patients even years after the end of oncology treatment [27,28]. Fear of movement has been identified as a barrier to exercise for patients with neuromusculoskeletal conditions but has been little explored in patients with BC [6,29]. Pain-related fear has been increasingly recognized as an important contributor to the maintenance of pain. Following oncology treatment and a prolonged period of rest during cancer treatment, survivors may perceive activity levels that were previously well tolerated as fatiguing. If due to increased fear levels, one of the responses is to avoid or limit PA levels, instead of gradually increasing them, then the fatigue may paradoxically persist and lead to a reduced quality of life [30,31,32]. Moreover, greater decreases in PA after BC diagnosis observed among heavier patients implies a potential for greater weight gain among women who are already overweight [9]. 

The aim of this study is to identify the level of kinesiophobia among women after BC (one year after surgical treatment, radiotherapy, or chemotherapy) depending on socio-demographic variables, stage and type of BC, lifestyle, and comorbidities. This study is important because kinesiophobia can create a hypokinetic attitude and lead to sedentary behaviors. 

## 2. Materials and Methods

### 2.1. Study Design 

In this cross-sectional study, 138 women participated one year after BC hospital treatment (surgical treatment, radiotherapy, or chemotherapy). Only women 18+ took part in the survey and they all had BC once. Some of the studied cancer survivors were still under antihormonal treatment, one of the longest examples of cancer treatment (usually lasting 5–10 years). 

The Tampa Scale of Kinesiophobia (TSK) questionnaire assessing the level of fear of movement due to expected pain and fatigue among cancer survivors was sent to the two leading patient organizations for women after BC in the Poznan district, Poland (Amazonki and Rakujemy jak chcemy). In this study, the Polish adaptation of the TSK scale in terms of pain and fatigue for cancer patients was used [33]—the TSK items used in this study are presented in the Supplementary File S1 and the characteristics of the surveyed patient organizations are presented in the Supplementary File S2. The reliability of the internal consistency of the scale was tested by examining the Cronbach’s alpha scores for each question (statement). The mean values for this indicator were 0.74 for the pain-related scale and 0.84 for the fatigue-related scale [33]. 

The questionnaire was sent by members of the management board to all members of patient organizations with a request to be filled in only by women who had already completed hospital treatment for a year. Women were informed about the aim of the anonymous and voluntary survey and that they could stop the questionnaire at any time. Responding to kinesiophobia questions was treated as informed consent to take part in the study.

### 2.2. Study Participants

Referencing a total possible population of 10,000 BC patients every year in Polish agglomerations, at a confidence level of 2σ of 95.5%, an error limit of 3%, and heterogeneity of 50%, I obtained a minimal representative sample of 111 women. That number is below 138, which is an obtained sample size (so the minimal sample was reached). The *Rakujemy jak chcemy* organization includes 100 women with BC, and the *Amazonki Poznań* organization includes 300 women with BC; thus, in this study 35% of BC survivors participated. At the Greater Poland Cancer Center in Poznan, 800 new cases of BC are diagnosed every year.

### 2.3. Research Tool—The Tampa Scale of Kinesiophobia (TSK)

The TSK used in this study is one of the most frequently cited measures for studying fear of movement. The TSK was introduced in 1991 in England by Miller [34]. The scale has been validated, adapted, and translated by many researchers, e.g., into Japanese [35], Portuguese [36], Spanish [37], Swedish [38], Dutch [30,31], and Polish [39], and used in assessing kinesiophobia in many diseases, e.g., rheumatoid arthritis [40], Parkinson’s disease [41], idiopathic scoliosis [42], multiple sclerosis [43], fibromyalgia [44], coronary artery disease [39], and myocardial infarction [45]. Kinesiophobia has been identified as a barrier to practicing sport for people with different diseases but has been minimally studied in patients with BC [7]. The TSK contains 17 items in which patients respond according to a four-point Likert scale, ranging from 1 to 4: “I do not agree completely”, “I do not agree in part”, “I agree partially”, and “I agree completely”. The scoring of questions 4, 8, 12, and 16 is reversed. The total score is in the range of 17–68 points. The higher the score, the greater the severity of the kinesiophobia. Researchers have developed a cutoff score and reported that patients who scored greater than 37 were high responders [23,24,25]. Similarly, this cutoff score was used to identify BC women with TSK and specifies fatigue [46] and pain [30,31]. Respondents answered questions that concerned, first pain, then fatigue.

During the survey, the participants were also asked to assess the level of anxiety before performing such physical activities recommended by WHO as brisk walking, running, swimming, cycling, aerobics, yoga, gym, etc.

The following [demographic] data from the respondents were also collected: level of education, place of residence, professional life, and marital status, as well as data about the stage (0,1,2,3,4) and type (luminal A/B, HER2+, triple negative) of BC. Women were also asked about their level of PA before the cancer diagnosis, actual comorbidities, and knowledge about WHO recommendations for cancer survivors.

### 2.4. Ethical Issues

The diagnostic survey among BC women using the TSK online questionnaire was approved by the Bioethics Committee at the Poznan University of Medical Sciences in Poland and was conducted from June 2022 to August 2022. According to the Committee’s decision, the study is not a medical experiment and thus can be conducted among women after BC.

### 2.5. Statistical Analysis

Descriptive data were expressed in % or mean ± SD. The Kolmogorov-Smirnov test was used to evaluate the distribution of normality. An unpaired t-test was also conducted to compare the demographic and clinical characteristics between the groups. Cohen’s d values were calculated to assess the effect size. Pearson’s chi-square test was used to analyze categorical data, and Spearman’s rank correlation coefficients were used to evaluate the relationship between the TSK scores and the rest of the variables. The Spearman’s rank correlation coefficients were accepted as follows: 0.81–1.0 excellent, 0.61–0.80 very good, 0.41–0.60 good, 0.21–0.40 fair, and 0–0.20 poor. A *p*-value of less than 0.05 was considered statistically significant. Statistical analysis was performed using IBM SPSS Statistics 28.0 (SPSS Inc., Chicago, IL, USA).

## 3. Results

### 3.1. Characteristics of the Surveyed BC Women

The mean age of the study respondents was 46.5 ± 9.2. The BMI—24.6 ± 4.0 among the studied women was very close to the barrier of overweight—25.0. The majority of BC women declared higher education (73.7%), living in large cities (50.4%), and being professionally active (65%). The studied women were mainly in either marriage or a relationship (78.1%)—Table 1.

### 3.2. The Level of Kinesiophobia among BC Survivors

Respondents declared significant levels of kinesiophobic pain and fatigue (both > 37 points). The general level of kinesiophobia—fatigue was 41.2 ± 6.2 and of kinesiophobia—pain was 41.3 ± 6.2.

### 3.3. Lifestyle of the BC Women

Almost half of the surveyed women after BC did not know the WHO recommendations regarding the weekly amount of PA for healthy people (47.8%) and for people with cancer (48.6%). Before the cancer diagnosis, 43.5% of the women did not practice a sport in accordance with the WHO recommendations. Every third woman after BC (32.6%) did not practice sport regularly one year after BC treatment. The results of the study (Table 2) show that the level of PA of the surveyed women and their knowledge of the WHO recommendations are still insufficient.

### 3.4. The Level of Kinesiophobia and Lifestyle before BC Diagnosis

The comparison of kinesiophobia one year after hospital treatment among women who were previously physically active (before the cancer diagnosis) and women who were not physically active was checked in order to see if the level of kinesiophobia was different in these groups (Table 3).

It turns out that the level of kinesiophobia differs significantly between women who were active before the cancer diagnosis and women who were not; therefore, previous lifestyle impacts the level of kinesiophobia after BC. Women who were not physically active before the BC diagnosis declared higher levels of kinesiophobia (42.8).

### 3.5. The Level of Kinesiophobia and Lifestyle One Year after BC Hospital Treatment

The study results show that a high level of kinesiophobia influences a low level of PA (43.2—kinesiophobia fatigue and 42.5—kinesiophobia pain among nonactive women one year after BC treatment)—Table 4.

### 3.6. The Level of Kinesiophobia According to Cancer Stage (Pre-Invasive & Early Stage vs. Late Breast Cancer)

In the next step, the relationship between the BC stage and the level of kinesiophobia was checked (Table 5), which indicates that the cancer stage does not influence the level of kinesiophobia.

### 3.7. The Level of Kinesiophobia According to BC Type (Luminal A & B vs. HER2+ vs. Basal/Triple Negative)

There are three main subtypes of BC: hormone-dependent (luminal), triple negative, and HER2 positive. As physical activity plays a particularly important role in the development of hormonally-dependent breast cancers, the level of kinesiophobia among women with hormonally-dependent luminal cancers has been checked (Table 6).

There were no statistically significant differences in the level of kinesiophobia depending on the type of BC. However, women with hormonally-dependent breast cancers should have lower levels of kinesiophobia in order to be physically active as often as possible.

### 3.8. The Level of Kinesiophobia According to Comorbidities

The next issue was to determine whether the coexistence of comorbidities affects the level of kinesiophobia (Table 7) because the main physiologic barriers to PA participation among oncology patients are cancer-related or treatment-related side effects (e.g., fatigue) and prevalent comorbidities.

The study results show that comorbidities can affect the level of kinesiophobia among women after BC. Significantly higher levels of kinesiophobia were declared by women with diabetes (50.6—kinesiophobia fatigue; 49.8—kinesiophobia pain) than by women without diabetes (41.0—kinesiophobia fatigue; 41.0—kinesiophobia pain). The existence of obesity also influences the level of kinesiophobia. Significantly higher levels of fear of movement were declared by women with obesity (47.8—kinesiophobia fatigue; 47.4—kinesiophobia pain) than by women of correct weight (40.5—kinesiophobia fatigue; 40.5—kinesiophobia pain).

### 3.9. The Level of Kinesiophobia According to Socio-Demographic Variables

A direct association between the level of kinesiophobia (pain and fatigue) and age has been found (Table 8), i.e., the greater the age, the higher the levels of kinesiophobia-pain and kinesiophobia-fatigue. No associations were found in the other socio-demographic variables.

## 4. Discussion

The aim of this study was to identify the level of kinesiophobia among women after BC (one year after surgical treatment, radiotherapy, or chemotherapy) depending on socio-demographic variables, stage and type of BC, lifestyle, and comorbidities. Polish BC survivors suffer from kinesiophobia significantly. Every third woman surveyed does not practice sport regularly one year after hospital treatment for BC. Their lifestyle before a BC diagnosis also impacts the level of kinesiophobia after treatment—women who were not physically active before the BC diagnosis declared higher levels of kinesiophobia than previously active women. According to this study, a high level of kinesiophobia influences a low level of PA among women after BC. Older women and women with comorbidities such as obesity and diabetes declare higher levels of kinesiophobia than women without comorbidities.

The rate of kinesiophobia in patients with BC in Turkey was found to be 30.8 [29], while in this study, the rate of kinesiophobia among women after BC in Poland is much higher (41.2; 41.3). This may result from less effective sports management in Polish oncology hospitals and physiotherapy centers, or it could have been influenced by the coronavirus pandemic.

BC women present with numerous side effects that may affect their quality of life. Exercise has been demonstrated to reduce some of these side effects, but few BC patients know and follow the exercise recommendations necessary to remain healthy [9,47]. In this study, almost half of the surveyed women after BC did not know the WHO recommendations regarding the weekly amount of PA for healthy people (47.8%) and for people with cancer (48.6%). Before cancer diagnosis, 43.5% of women did not practice sport in accordance with the WHO recommendations. Every third woman after BC (32.6%) failed to practice sport regularly one year after BC treatment. The results of the study showed that the level of PA among the surveyed women, and their knowledge of the WHO recommendations, are still insufficient, even though the majority of the surveyed women have higher education (73.7%).

A qualitative study carried out by Sander et al., 2012 showed that oncology patients who were physically active before BC diagnosis are not afraid to exercise [7]. Also in this study, BC patients who were physically active before diagnosis declared lower levels of kinesiophobia than women who were not active before oncology treatment. The study results also show that a high level of kinesiophobia correlates with a low level of PA one year after BC treatment. Unfortunately, kinesiophobia may increase the risk for lymphedema and depression/anxiety and decrease upper extremity functioning in BC survivors.

According to Irwin et al., greater decreases in PA are observed among obese patients when compared with patients of normal weight [9]. In this study, women with diabetes and obesity declared higher levels of kinesiophobia than women without comorbidities. Physical movement is extremely important in chronic diseases. Women with abnormal body weight show fear of movement because they may become tired quickly or have difficulty performing certain body movements, while women with diabetes may experience the effects of diabetic polyneuropathy, among other disorders. Efforts to encourage and facilitate PA among BC women would be an important tool to decrease obesity, prevent post-diagnosis weight gain, and improve BC prognosis. Previous studies have found that diabetes was associated with a close to 40% increase in mortality within the first 5 years following BC—early survival following BC is reduced in women with diabetes, possibly due to diabetes-related causes [48], and systematic PA is also extremely important in diabetics who do not suffer from BC.

In other studies of kinesiophobia, age does not differentiate the level in patients with multiple sclerosis or Parkinson’s disease or among people who have suffered a stroke. However, in the case of multiple sclerosis, there is a slight tendency towards an increase in the level of kinesiophobia noticeable with the patients’ age and the duration of the disease. Place of residence did not differentiate kinesiophobia in people with Parkinson’s disease and after a stroke; however, it did differentiate kinesiophobia in people suffering from multiple sclerosis—living in larger cities was associated with a lower level of kinesiophobia compared to inhabitants of villages and small towns. Level of education and marital status did not differentiate kinesiophobia in people with Parkinson’s disease, multiple sclerosis, or after a stroke [26,43]. This study shows that age differentiates kinesiophobia in female oncology—the greater the age, the higher the levels of kinesiophobia among BC survivors. No associations were found in the other socio-demographic variables.

There were no statistically significant differences in the level of kinesiophobia depending on the stage and type of BC. However, women with hormonally dependent breast cancers should have lower levels of kinesiophobia in order to be physically active as often as possible because PA plays a particularly important role in the development of hormonally dependent breast cancers. If the level of kinesiophobia does not differ depending on the type of BC, this is probably due to a lack of knowledge on the part of patients that each type has its own properties, and that doctors should encourage PA especially for patients with hormone-dependent cancer. Research shows that the level of kinesiophobia is the same regardless of the degree of BC diagnosed. A common misconception among less-informed patients suggests that exercise may promote the spread of neoplastic cells, so perhaps patients with early BC are afraid of metastases. On the other hand, patients in later stages—that is, with metastases—including bone metastases that are very common in BC—may, for example, be afraid of falling due to advanced osteoporosis.

BC is increasingly becoming a chronic rather than a fatal disease. It is, therefore, important to educate women that it is possible to live actively after cancer [12]. In the future, cancer patients should also be examined during radiotherapy, hormone therapy, immune therapy, and chemotherapy because each type of treatment creates different conditions for exercising PA. People suffering from malignancies other than BC should also be examined (PA is also important in prostate or colon cancer).

Coping with kinesiophobia after cancer is a dynamic process that requires both internal and external support. The problem of kinesiophobia requires an individualistic approach (knowledge about socio-demographic variables, comorbidities, and lifestyle before diagnosis) and broad-based government strategies. It is also extremely important to consider the factors specific to cancer sites and treatments in order to improve PA participation and motivation for sports in cancer survivors. Moreover, affordable, accessible, and inclusive community-based fitness facilities, with sports instructors knowledgeable about cancer and preventative treatments, are needed in modern society in order to overcome the barriers to PA participation among cancer survivors. Exercise should be an integrative complementary intervention to improve physiological, physical, and psychological factors that affect survival and quality of life among women with BC.

The main strength of this study is the large number of women after BC who participated in the survey (138 from 400 united in the patient organizations). The main limitation is that the study involved women associated with patient organizations, which usually organize leisure time for cancer patients in the form of sports activities or educational programs. Women who were not members of those organizations and who did not participate in this survey potentially show even lower interest in PA and a higher level of kinesiophobia than the respondents who took part. Moreover, this study is cross-sectional; it would be worthwhile also to conduct a longitudinal study in the future to observe how the attitudes of oncology patients towards PA change during several years of treatment.

## 5. Conclusions

BC survivors still suffer from kinesiophobia one year after finishing oncology treatment. Every third woman with BC does not practice sport regularly. Lifestyle before cancer impacts the level of kinesiophobia after treatment; a high level of kinesiophobia influences a low level of PA. Older women and women with comorbidities like obesity and diabetes declare higher levels of kinesiophobia than women without comorbidities. Unfortunately, low PA after BC observed among heavier patients implies a potential for greater weight gain among women who are already overweight. Women with abnormal body weight show fear of movement because they potentially tire quickly and may have difficulty performing certain body movements, and women with diabetes may experience the effects of diabetic polyneuropathy, among other disorders.

The results of this study indicate that rehabilitation and recovery programs must take into account an individual approach to the oncological patient (for example, knowledge about age, current lifestyle, or comorbidities) and should also take into account the history of lifestyle before the disease. Regular PA should be treated by patients and doctors as a permanent and important element of the treatment and prevention of disease recurrence. During the COVID-19 pandemic, not all patients could benefit from rehabilitation programs due to fear of infection in hospitals. People who live far from oncology centers also often fall out of the habit of attending rehabilitation programs. Therefore, it seems that the role of educators in postoperative care in promoting a healthy lifestyle should be taken over by smaller local health centers and gym instructors.

## Figures and Tables

**Table 1:** Characteristics of the respondents (*n* = 138).

	SD, %
Age	46.5 ± 9.2
Body mass index	24.6 ± 4.0
Achieved university education (or still a student)	73.7%
Achieved secondary level of education	26.3%
Living in a village	19.7%
Living in a small city (less than 20 000 inhabitants)	8.8%
Living in a medium city (20–100 000 inhabitants)	21.2%
Living in a large city (more than 100,000 inhabitants)	50.4%
Professionally active	65.0%
Pensioner (age reason)	8.0%
Pensioner (health reason)	18.2%
Student	0.7%
Unemployed	8.0%
In marriage/relationship	78.1%
Single	7.3%
Widowed or divorced	14.6%

Data are shown as mean ± standard deviation (SD) and percentages (%).

**Table 2:** Lifestyle of the BC women.

	SD, %
Did you know the WHO recommendations for healthy adults?	Yes: 52.2% No: 47.8%
Do you know the WHO recommendations for people with cancer?	Yes: 51.4% No: 48.6%
Did you lead an active lifestyle before the BC diagnosis? (in accordance with WHO recommendations presented by the researcher during this study)	Yes: 56.5% No: 43.5%
For how many years have you been physically active? (in accordance with the WHO recommendations)	8.4 ± 11.2 *
For how many years have you not been physically active? (not in accordance with WHO recommendations)	4.4 ± 9.7 *
Do you lead an active lifestyle now after oncology treatment? (in accordance with WHO recommendations)	Yes: 67.4% No: 32.6%

* Data are shown as mean ± standard deviation (SD) and percentages %.

**Table 3:** The impact of lifestyle before BC diagnosis on the level of kinesiophobia one year after hospital treatment.

	Women Physically Active before Treatment *n* = 78	Women Not Physically Active before Treatment *n* = 60	*p*	*Cohen’s d*
Average Kinesiophobia Fatigue	40.0 ± 6.0	42.8 ± 6.1	0.004	6.050
Average Kinesiophobia Pain	40.0 ± 6.1	42.8 ± 6.1	0.005	6.105

Data are shown as mean ± standard deviation (SD).

**Table 4:** Impact of kinesiophobia on lifestyle one year after hospital treatment.

	Women Physically Active Now *n* = 93	Women Not Physically Active Now *n* = 45	*p*	*Cohen’s d*
Average Kinesiophobia Fatigue	40.3 ± 6.0	43.2 ± 6.1	0.005	6.060
Average Kinesiophobia Pain	40.6 ± 6.1	42.5 ± 6.3	0.045	6.193

Data are shown as mean ± standard deviation (SD).

**Table 5:** The level of kinesiophobia and BC stage.

	Pre-Invasive and Early Breast Cancer (Stages: 0,1,2) *n* = 87	Late Breast Cancer (Stages: 3,4) *n* = 51	*p*	*Cohen’s d*
Average Kinesiophobia Fatigue	41.5 ± 6.6	40.7 ± 5.4	0.211	7.606
Average Kinesiophobia Pain	41.6 ± 6.7	40.7 ± 5.1	0.198	7.482

Data are shown as mean ± standard deviation (SD).

**Table 6:** The level of kinesiophobia and type of BC.

	Luminal (A + B) *n* = 86	HER2+ *n* = 34	Basal/Triple Negative *n* = 18	*p* Value Inter-Groups	F Value	Effect Size
Average Kinesiophobia Fatigue	41.0 ± 5.9	41.1 ± 7.3	42.3 ± 5.5	0.710	0.344	0.005
Average Kinesiophobia Pain	41.2 ± 6.0	40.4 ± 7.2	42.9 ± 5.5	0.381	0.973	0.014

Data are shown as mean ± standard deviation (SD).

**Table 7:** The level of kinesiophobia and comorbidities.

	Average Kinesiophobia Fatigue	*p*	* Cohen’s d *	Average Kinesiophobia Pain	*p*	* Cohen’s d *
Without diabetes	41.0 ± 5.9	<0.001	6.091	41.0 ± 5.9	0.001	6.132
With diabetes	50.6 ± 10.3			49.8 ± 10.8		
Without hypertension	41.4 ± 6.6	0.499	6.415	41.4 ± 6.7	0.497	6.404
With hypertension	41.1 ± 5.2			41.4 ± 4.9		
Without obesity	40.5 ± 5.6	<0.001	5.933	40.5 ± 5.5	<0.001	5.969
With obesity	47.8 ± 8.1			47.4 ± 8.7		
Without osteoporosis	41.1 ± 6.0	0.084	6.359	41.1 ± 6.0	0.066	6.337
With osteoporosis	43.8 ± 8.6			44.0 ± 8.6		

Data are shown as mean ± standard deviation (SD).

**Table 8:** Correlation of kinesiophobia scores with sociodemographic variables.

	Kinesiophobia Fatigue		Kinesiophobia Pain	
	r	*p*	r	*p*
Level of education	−0.094	0.275	−0.071	0.410
Place of residence	0.146	0.089	−0.130	0.131
Marital Status	0.038	0.658	0.037	0.664
Occupational Status	0.101	0.243	0.132	0.125
Age	0.192	0.025	0.173	0.043

r: Spearman’s rank correlation coefficients, accepted as follows: 0.81–1.0 excellent, 0.61–0.80 very good, 0.41–0.60 good, 0.21–0.40 fair, and 0–0.20 poor.

## Data Availability

The raw data supporting the conclusions of this article will be made available by the author without undue reservation.

## Supplementary Materials

The following supporting information can be downloaded at: https://www.mdpi.com/article/10.3390/ijerph192114565/s1.
