# Supplementary material for: Kinesiophobia among Breast Cancer Survivors One Year after Hospital Treatment
Source: Int J Environ Res Public Health. 2022 Nov 6;19(21):14565. doi: 10.3390/ijerph192114565 (PMC9655552; doi:10.3390/ijerph192114565)
Supplement: Supplementary file 1 [file ijerph-19-14565-s001.zip › ijerph-2017501-File S2.pdf]

Supplementary File S2:

Rakujemy jak chcemy Association is an organization operating in Poznań, Poland, dedicated to the idea of helping people suffering from BC. They work with full dedication to make the path of girls with cancer and all those in need easier. Their goals include: Running a support group on Facebook, Hosting webinars with treatment experts and more, Meeting patients both in the hospital and at their headquarters, Cooperation with local foundations and patient organizations, Organizing girls' social meetings during and after oncological treatment, Promotion and education about the latest therapies and treatment methods. The aim of the *Amazonki Organization* is to represent the interests of women after BC treatment and full psychophysical rehabilitation.
